# Strategies for enhancing delivery efficiency on MR‐Linac: A dosimetric study and historical plan review

**DOI:** 10.1002/acm2.70638

**Published:** 2026-05-27

**Authors:** Ying Zhang, Shanshan Tang, Christopher Kabat, Justin Visak, Tsuicheng Chiu, Ruiqi Li, Steve Jiang, Mu‐Han Lin

**Affiliations:** ^1^ Medical Artificial Intelligence and Automation (MAIA) Lab & Department of Radiation Oncology UT Southwestern Medical Center Dallas Texas USA

**Keywords:** delivery efficiency, MR‐Linac, online adaptive radiotherapy, optimal planning

## Abstract

**Purpose:**

The Elekta Unity MR‐Linac enables online adaptive radiotherapy (oART) with integrated 1.5T MRI. Given its relatively low dose rate, identifying optimal IMRT planning parameters is essential to achieve efficient delivery. This study investigates parameter optimization strategies that enhance delivery efficiency while maintaining high dosimetric quality.

**Methods:**

A dosimetric study was designed using ten prostate simultaneous integrated boost (SIB) patients (45 Gy/40 Gy in five fractions) and 10 liver SBRT patients (42–54 Gy in 3–5 fractions). Each patient has three plans: (1) Clinical, (2) Refa (segment‐limited, ≤120 segments), and (3) Refb (MU‐modulated plan with larger minimum segment width (0.7 cm or 1 cm) and higher fluence map smoothing level (medium or high)). A linear equation for estimating delivery time was established and validated against 1173 retrospective Unity plans based on total segments and Monitor Units (MUs). All the study plans were evaluated by comparing delivery efficiency related metrics (total number of segments, MUs, estimated delivery time), and plan quality using dose–volume histogram (DVH) metrics for targets and organs‐at‐risk (OARs). Additionally, an online adaptive study was performed on representative cases: the three reference plans were adapted to a daily image, and the resulting adaptive plans were assessed for optimization time and plan quality against clinical criteria.

**Results:**

Using the derived delivery time estimation equation on 173 unseen test cases, the mean absolute error (MAE) between estimated and recorded times was 0.9 min, with 84% of cases with a prediction error within ±1 min, and with 95% of cases falling within ±2 min. Both strategies reduced delivery complexity compared with clinical plans. For prostate cases, Refa and Refb lowered segment counts to <120 (vs. original 137–178) and reduced MUs from 3022 to 2899 (Refa) and 2497 (Refb), respectively. Delivery times decreased on average by 2.6 min with Refa (up to 5.4) and 4.0 min with Refb (up to 6.7). For liver cases, segment counts dropped from (149–198) to <120, with mean MUs reduced from 4233 to 3528 (Refa) and 3309 (Refb). Delivery times were shortened from 24.4 min to 18.9 (Refa) and 18.3 (Refb), up to 7.6 min saving for Refa and up to 8.9 min for Refb. Across both cohorts, PTV coverage and OAR sparing were maintained, with no statistically significant differences (*p* > 0.05). All adaptive plans from Refa and Refb were completed within 5–13 min, consistent with standard clinical online optimization times. After normalization to the clinical adaptive plan, all plans met the dosimetric goals. A similar time‐saving trend was observed across the adaptive plans.

**Conclusions:**

Systematic adjustment of Unity IMRT planning parameters—specifically limiting total segment number, reasonable minimum segment width, and fluence smoothing—can markedly improve delivery efficiency while maintaining clinically acceptable dosimetric quality. These findings provide practical, evidence‐based guidelines for parameter selection in Unity planning, supporting reduced treatment times, improved patient throughput, and broader clinical feasibility of MR‐guided online adaptive radiotherapy.

## 1 INTRODUCTION

The Elekta Unity MR‐Linac integrates a 1.5 Tesla Magnetic Resonance Imaging (MRI) scanner with a linear accelerator,^1^ allowing for daily adjustment of treatment plans based on the patient's anatomy as observed on the MRI at the time of treatment.^2^ This enhanced precision and adaptability offer significant clinical benefits but also introduce considerable challenges—most notably, prolonged treatment session durations. Adaptive treatments, particularly those involving the “Adapt to Shape” (ATS) workflow, are resource‐intensive, requiring extended time for imaging, contouring, re‐planning, and verification—all performed while the patient remains on the treatment table. These sessions can last between 60 and 120 min when a full clinical team is engaged.^3^, ^4^ Recent analyses show that the median ATS workflow duration is 34 min, with plan adaptation (including re‐contouring and re‐planning) accounting for 27% of the session and beam delivery making up 40%.^5^ AI‐based auto‐segmentation tools are increasingly being integrated into clinical practice to enhance contouring efficiency.^6^, ^7^ Since adaptive workflows already prolong treatment sessions, minimizing beam‐on time is especially important. Efficient radiation delivery is essential for maintaining patient comfort, reducing intrafraction motion,^8^ and for enhancing throughput on the Elekta Unity system.

Although the Elekta Unity uses a 7MV flattening filter‐free (FFF) beam, it operates at a fixed dose rate of nominal 425 monitor units per minute (MU/min)—significantly lower than other FFF‐capable systems, which can exceed 1200 MU/min.^9^ This hardware constraint creates a bottleneck in the clinical workflow, as the dose rate directly impacts radiation delivery speed. Despite the Unity's fast 6 RPM gantry and high‐speed MLCs, these mechanical advantages cannot fully offset the limitations imposed by the capped dose rate when high MUs are required. Plans with high MU counts not only extend delivery time but may also increase out‐of‐field dose and accelerate machine wear. As a result, MU reduction is a key strategy for improving delivery efficiency. Additionally, the number of segments in step‐and‐shoot intensity‐modulated radiation therapy (IMRT) significantly influences delivery time. A higher number of segments generally prolongs treatment delivery due to increased MLC movements and beam‐on/off cycles. Specifically, for step‐and‐shoot IMRT on the Elekta Unity, an inter‐segment pause contributes to the overall treatment duration. This latency between beam‐on and beam‐off for each segment makes minimizing the total number of segments a critical factor for delivery efficiency. For unity, auto field sequence (AFS) allows a series of beams to be delivered in a single automated sequence, avoiding the significantly more time‐consuming beam‐by‐beam loading process. However, there is a limitation on the maximum number of segments per AFS, with each AFS supporting fewer than 125 segments. When the total number of segments exceeds this limit, two AFS are typically required. The termination and relaunch of an additional AFS can take several extra minutes, thereby prolonging the overall treatment delivery time.

The clinical introduction of Comprehensive Motion Management (CMM)^10^ on MR‐Linac platforms further underscores the importance of optimizing plan‐delivery parameters. Efficient delivery not only supports accurate dose accumulation under motion monitoring but also ensures that overall machine throughput can be maintained as adaptive case volume expands. Streamlined planning and delivery parameters help minimize beam‐on time while preserving dosimetric robustness, enabling clinics to sustain a high level of adaptive service without compromising treatment quality or patient comfort. This proactive optimization aligns with the broader goal of making adaptive radiotherapy both clinically effective and operationally scalable.

IMRT achieves highly conformal dose distributions through an inverse planning process, in which beam intensities and machine parameters are iteratively optimized by algorithms to meet prescribed target coverage and organ‐at‐risk (OAR) dose constraints. While multiple IMRT parameter configurations can achieve clinically acceptable objectives, variations in these parameters may significantly influence treatment delivery efficiency, dosimetric quality, and their stability in the context of online adaptation. A systematic understanding of these effects is particularly important for optimizing the planning process on the Elekta Unity MR‐Linac platform. At our institution, treatment planning for the Elekta Unity is conducted by a dedicated team of over ten physicists, with experience levels ranging from senior specialists to junior practitioners. While standardized physician directives and uniform DVH criteria are systematically applied to ensure all clinical plans meet evaluation standards, significant inter‐planner variation remains. We observed that individual differences in IMRT optimization strategies, constraint selection, and planning parameters persist despite consistent clinical objectives. Although these variations do not compromise clinical acceptability, they introduce fluctuations in plan modulation and delivery time, serving as the primary motivation for this research. In addition, conservative planning strategies are often employed to ensure both plan quality and patient safety. For example, when sparing critical OARs such as the urethra, plans may be generated with a high number of segments and strict multileaf collimator (MLC) sequencing constraints, which can improve dosimetric outcomes at the expense of delivery efficiency.

The purpose of this study is to retrospectively review historical Elekta Unity IMRT plans generated at a single institution, with specific emphasis on delivery efficiency, plan complexity, and optimization parameter settings. In addition, dosimetric experiments were designed to systematically evaluate the impact of selected IMRT parameters on delivery efficiency. Based on these analyses, we provide practical recommendations for parameter selection to improve delivery efficiency on Unity and share institutional experience with the broader community.

## 2 METHODS

### 2.1 Delivery time estimation

A retrospective analysis was conducted to assess plan delivery efficiency. Basic plan parameters, specifically the total number of segments and total MUs, were extracted from 1000 online plans delivered between October 1, 2024, and February 28, 2025. Concurrently, process time for each step of online adaptation was retrieved from our in‐house dashboard, including the total delivery, from beam on to the end of the treatment. Linear fitting was performed using these data to develop an equation for estimating plan delivery time using total segment and MUs. The derived equation was subsequently validated using an additional dataset of 173 plans delivered between March 1 and April 1, 2025. The selected time period preceded the clinical implementation of CMM [10] at our institution, thereby eliminating the variable of prolonged delivery time due to CMM gating.

### 2.2 Dosimetric experiments

**FIGURE 1:**
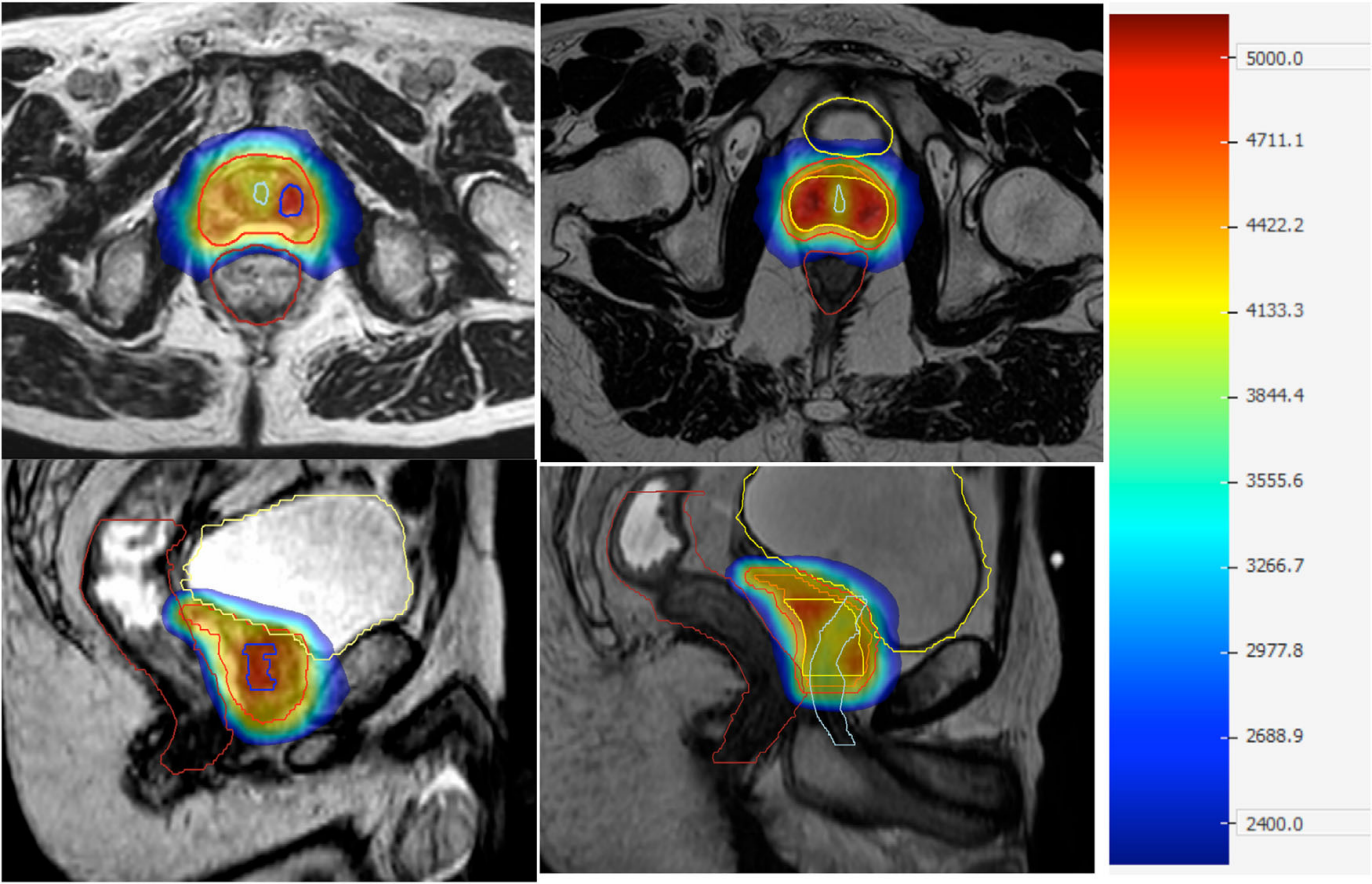
Representative examples of (top row) whole‐prostate boost SIB and (bottom row) micro boost SIB prostate case both with a prescription of 4500/4000 cGy in five fractions. The dose color wash is displayed between 2400 and 5000 cGy.

**FIGURE 2:**
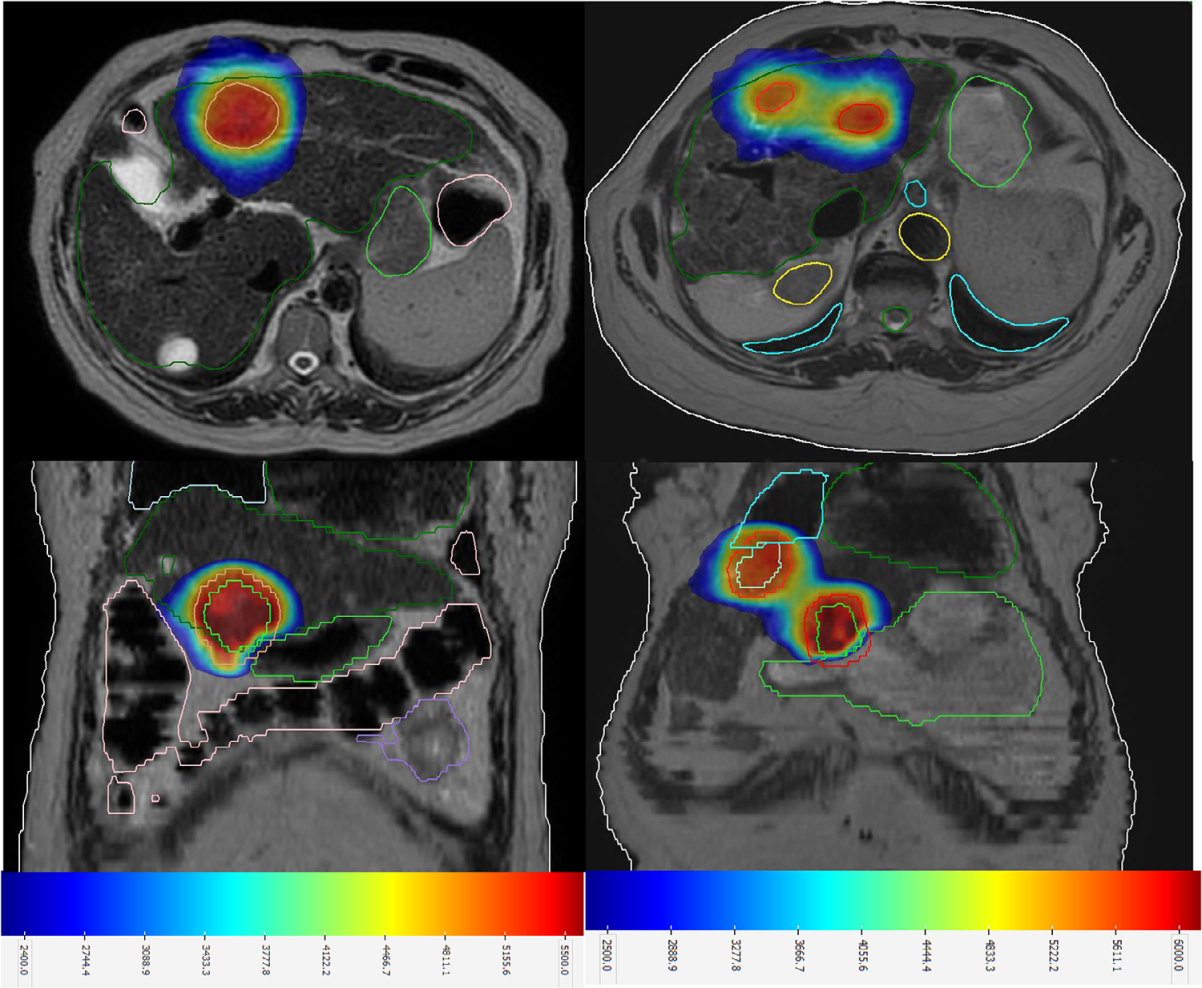
Representative liver examples showing (Left) a single‐lesion case with a prescription of 4800 cGy in three fractions and (Right) a two‐lesion case with a prescription of 5000 cGy in five fractions. The dose color wash is displayed between 2400/2500 cGy and 5500/6000 cGy.

At our institution, the Elekta Unity MR‐Linac has been employed for treatment across nearly all body sites. For the present study, we focused on prostate genitourinary (GU) and liver cases, with 10 patients selected for each site. All the patients were treated between October 2023 to April 2025. All prostate cases were treated with a simultaneous integrated boost (SIB) prescription of 45 Gy /40 Gy in five fractions. The primary target volume (40 Gy) encompassed the entire prostate, with a boost directed either to a micro‐lesion identified using multi‐sequence MRI or to the prostate excluding critical OARs (as shown in Figure 1). Rectal spacer gel was routinely used for prostate patients. PTV 45 Gy was defined with 2 mm margins from the urethra and bladder, and 0 mm from the rectum for OAR sparing. Liver cases demonstrated greater prescription variability, ranging from 42–54 Gy in 3–5 fractions depending on tumor size and anatomical location (as shown in Figure 2).

To enhance delivery efficiency, this study focuses on two primary objectives: (1) reducing the total number of segments and (2) modulating the total Monitor Units (MUs). Based on the our clinical planning experience, we focused on three important variables that have high impact on the final MUs: maximum segment count, fluence map smoothing level, and minimum leaf width. For each patient, three distinct ATS scenarios were defined and compared:

(1) Clinical pan: The original clinical plan used for treatment, typically utilizing a minimum leaf width of 0.5 cm and low‐to‐medium fluence smoothing with no strict cap on segment count. For all clinical plans, the number of beams ranged from 11–14. Plans were optimized with a small minimum segment width (0.5 cm), permitting higher modulation. Fluence map smoothing constraints varied between medium (“m”) and low (“l”) levels. The maximum allowed number of segments ranged from 150–179. For liver cases, similar parameters were applied: a minimum segment width of 0.5 cm (with one case at 0.7 cm and two cases at 1.0 cm for larger target volumes), smoothing set to medium or low, and maximum segment counts ranging from 150–200.

(2) Plan Refa (Segment‐Limited): The maximum number of segments was strictly capped at 120, while all other optimization parameters (fluence smoothing and minimum leaf width) were kept identical to those of the Clinical Plan. The selection of 120 segments was guided by the technical constraint of the Auto Field Sequence (AFS), which supports fewer than 125 segments per sequence. Setting the limit at 120 provides a practical buffer, allowing flexibility for potential adjustments during online adaptation while remaining within the single‐AFS capacity.

(3) Plan Refb (MU‐Modulated): In addition to capping the maximum segments at 120, delivery complexity was further reduced by increasing the minimum segment width (to 0.7 cm or 1.0 cm) and applying higher fluence smoothing levels (medium or high) compared to the Clinical Plan.

As all clinical plans were generated by various planners over time and our standard planning templates have been updated, the IMRT constraints used in the original plans varied. For plan comparison purpose, the same IMRT constraints used in the clinical plans were applied for specific case. Only minor adjustments were made for the experimental plans when necessary to meet specific clinical dosimetric criteria. Following optimization, all plans were normalized to achieve equivalent target coverage. For multiple‐lesion liver cases, normalization was performed to match the coverage of the worst‐covered lesion, consistent with the clinical planning approach. Additional details of the dosimetric study and delivery efficiency analysis are provided in the Supplemental Materials, including Table S1 summarizing plan characteristics and Table S2 summarizing delivery efficiency metrics for the initial reference plans and adaptive plans.

### 2.3 Performance evaluation

The three plan types‐Clinical, Ref‐a, and Ref‐b‐were systematically compared with respect to the actual number of deliverable segments, total monitor units (MUs), estimated treatment delivery time (calculated using a previously derived fitting equation) and achievement of clinical objectives. For prostate cases (*N* = 10), plan quality was assessed by comparing key dose–volume histogram (DVH) metrics for the planning target volumes (PTVs) and critical organs‐at‐risk (OARs). We evaluated coverage for both PTV45Gy and PTV40Gy, as well as OAR metrics including urethral sparing (D0.035cc < 42.8 Gy), rectal wall dose (D0.035cc < 45 Gy, V24Gy < 50%), and bladder wall dose constraints (D0.035cc < 42.8 Gy, V18.3 Gy < 18%). For liver cases (*N* = 10), analysis included both the coverage for internal target volume (ITV) and PTV. Liver sparing was quantified using the percentage of normal liver volume (Liver—ITV) receiving more than 21.5 Gy for 5 fractions and 17.7 Gy for 3 fractions. Maximum point doses (D0.035cc) to the stomach, esophagus, and heart, were also evaluated.

In addition, an online adaptive study was performed for selective cases using the offline Monaco planning system. To accurately mimic the online adaptive process, Refa and Refb plans were first approved and then adapted to a daily image. For prostate cases, one Adapt to Position (ATP) and three Adapt to Shape (ATS) scenarios were investigated. For liver cases, three cases with various PTV size (32.7cc single lesion, 84.5cc two lesions and 128cc single lesion) were used to generate ATS plans. The study evaluated both the optimization time for the three adaptive plans and the plan quality in terms of meeting clinical goals.

## 3 RESULTS

### 3.1 Delivery time estimation fitting

**FIGURE 3:**
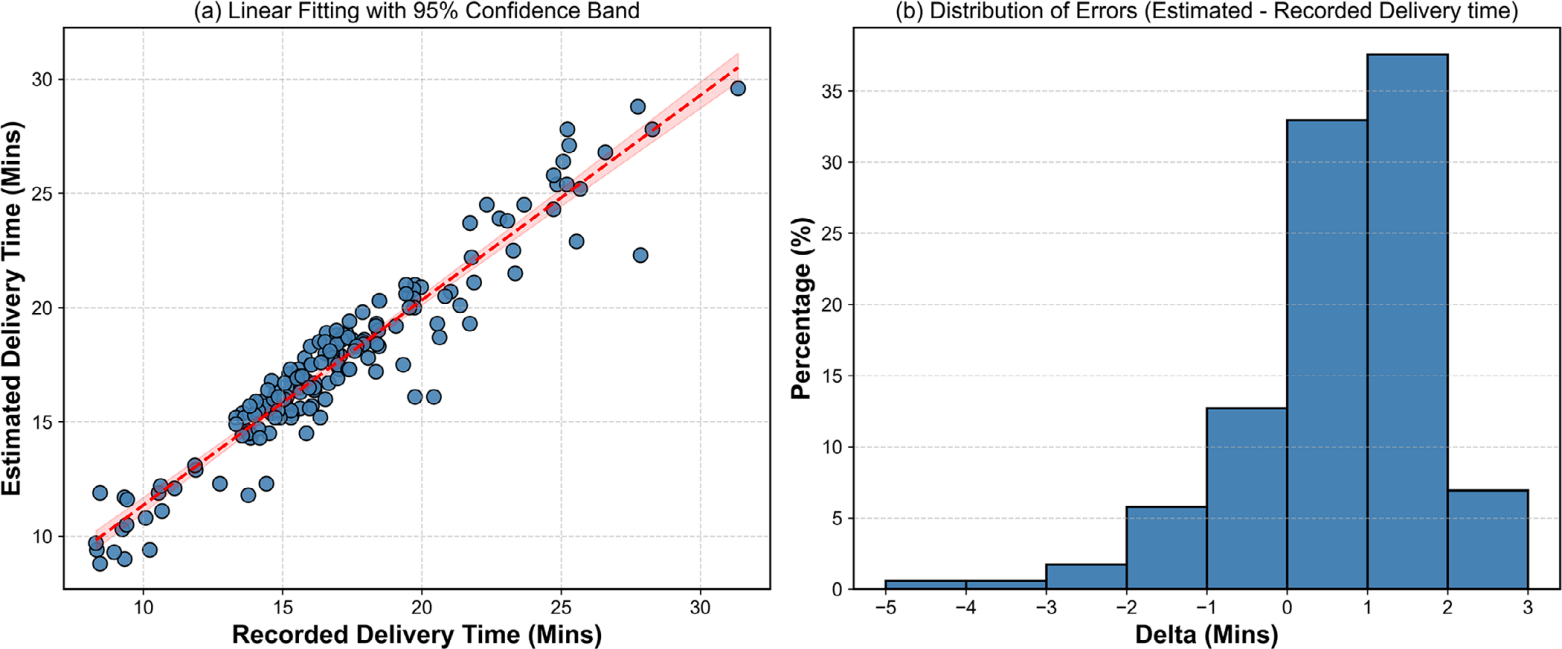
Validation of the delivery time estimation model. (a) Correlation between recorded and model‐estimated delivery times for an independent test set (*n* = 173). The red dashed line represents the linear fit (*R* ^2^ = 0.913), with the shaded pink region indicating the 95% confidence band. (b) Distribution of prediction residuals Delta = estimated—recorded time, demonstrating that the majority of errors fall within a ± 2 min window.

A linear regression model was developed to estimate delivery time as a function of the number of segments and total MUs:

$$\begin{array}{cc} \mathrm{Deliver}\;\mathrm{Time}\;\left(\mathrm{mins}\right) & =0.05966\times \mathrm{Total}\;\mathrm{Segments}\\ & \;+0.00307\times \mathrm{Total}\;\mathrm{MUs}+1.39 \end{array}$$

with a coefficient of determination 𝑅^2 ^= 0.74. Figure 3 presents the validation results for 173 independent testing plans that were not used during model training. The scatter plot (Figure 3a) demonstrates strong linear agreement between estimated and recorded delivery times. The 95% confidence band (shaded) is notably narrow in the 10–25‐min range, indicating high precision and robustness of the estimates, and widens slightly outside this interval due to fewer data samples. To further quantify model reliability, an Ordinary Least Squares (OLS) analysis was performed to estimate the standard errors (SE) of the linear regression parameters, yielding a slope of 0.897 ± 0.021 (SE) and an intercept of 2.63 ± 0.37 (SE). Figure 3b shows the corresponding error distribution (Δ = estimated − recorded time). The model demonstrates high predictive accuracy on this unseen testing dataset, with a mean absolute erroMean Absolute Error (MAE) of 0.9 min. Quantitatively, 84% of testing cases had a prediction error within ±1 min, and 95% fell within ±2 min.

### 3.2 Delivery time comparison

**FIGURE 4:**
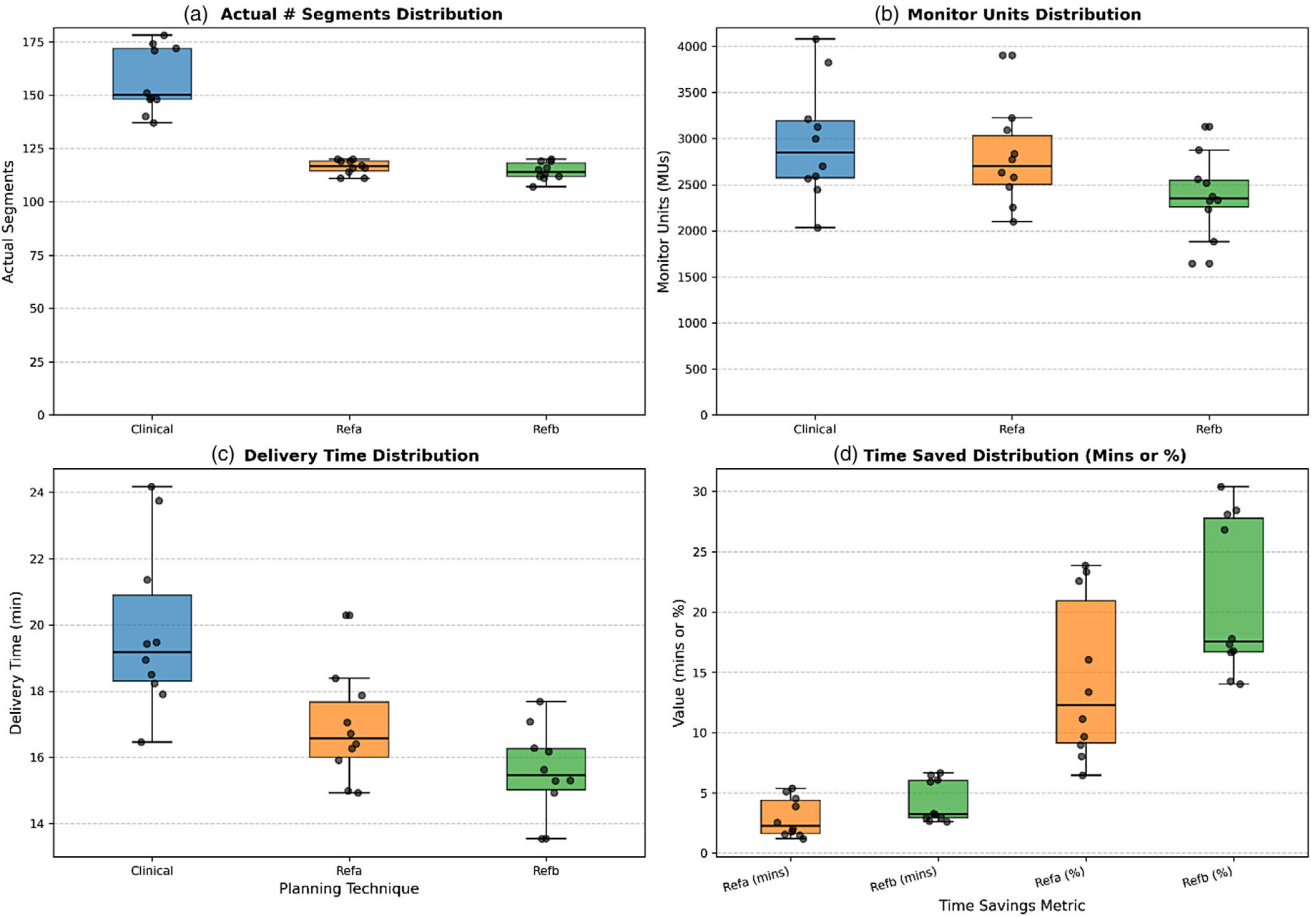
Comparison of clinical and experimental plans (Refa and Refb) for prostate cases. (a) Distribution of the actual number of segments. (b) Distribution of total monitor units (MUs). (c) Estimated delivery time distribution. (d) Distribution of delivery time savings in both minutes and percentage. Box plots represent interquartile ranges with whiskers denoting 1.5× IQR, and individual points indicate outliers.

As shown in Figure 4, for prostate SIB cases, the optimization strategies Refa and Refb consistently produced plans with fewer segments, lower monitor units (MUs), and substantially reduced estimated delivery times compared to the Clinical plans. Clinical plans used an average of 159.8 segments (range: 137–178), whereas Refa and Refb restricted the number of segments to fewer than 120, enabling delivery within a single AFS. Total MUs were also reduced in the optimized plans: Refa averaged 2987.8 MUs (versus 3156.0 for Clinical plans), while Refb achieved the greatest reduction, averaging 2464.4 MUs. These reductions in segments and MUs translated into significant delivery time savings. Clinical plans had an average estimated delivery time of 20.68 min. Refa plans showed an average time saving of 2.6 min per fraction, reaching up to 5.4 min for some cases, corresponding to a mean reduction of 19.1% and up to 23.9% reduction in delivery time. Refb plans were the most efficient, with an average delivery time of 15.64 min, yielding a mean time saving of 4 min per fraction and up to 6.7 min, representing an average of 19.2% and up to 30.4% reduction compared to Clinical plans.

**FIGURE 5:**
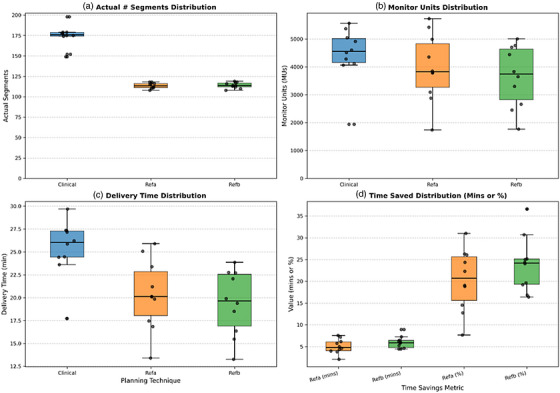
Comparison of clinical and experimental plans (Refa and Refb) for Liver cases. (a) Distribution of the actual number of segments. (b) Distribution of total monitor units (MUs). (c) Estimated delivery time distribution. (d) Distribution of delivery time savings in both minutes and percentage. Box plots represent interquartile ranges with whiskers denoting 1.5× IQR, and individual points indicate outliers.

Similarly, as in Figure 5, both the Refa and Refb improved plan efficiency for Liver cases. The Clinical plans for Liver treatments averaged 168.7 segments (range: 149–198), while the optimized plans reduced this to an average of 112 segments (Refa) and 114 segments (Refb), enabling one AFS delivery. The average MUs were 4232.6 for Clinical plans. The Refa plans averaged 3528 MUs, and Refb plans averaged 3309 MUs. The average estimated Delivery Time for Clinical Liver plans was 24.4 min. Both optimized strategies provided substantial time savings: Refa plans had an average delivery time of 18.9 min, resulting in a mean time saving of 5.5 min per fraction. This represents an average reduction of 22.9% and up to 31% in delivery time. Refb plans achieved an average delivery time of 18.3 min, resulting an average time saving of 6.1 min per fraction, reaching up to 8.9 min for some cases, corresponding to a mean reduction of 25.3% and up to 36.6% reduction in delivery time.

### 3.3 Dosimetric plan quality comparison

**FIGURE 6:**
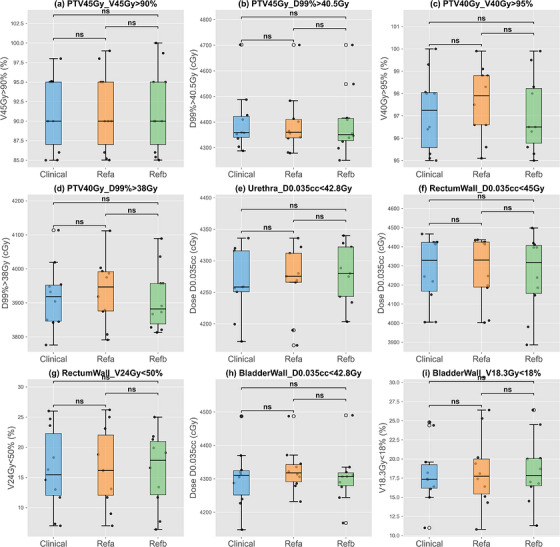
Comparison of key plan quality metrics for clinical and experimental (Refa and Refb) plans in prostate cases. Metrics are presented as (a) PTV45Gy, V45Gy > 90%, (b) D99% > 40.5 Gy, (c) PTV40Gy, V40Gy > 95%, (d) D99% > 38 Gy, (e) urethra D0.035 cc < 42.8 Gy, (f) rectal wall D0.035 cc < 45 Gy and (g) V24Gy < 50%, (h) bladder wall D0.035 cc < 42.8 Gy and (i) V18.3 Gy < 18%. The connecting lines between the boxplots indicates pairwise *t*‐tests, with “NS” denotes no statistically significant difference (*p* > 0.05).

**FIGURE 7:**
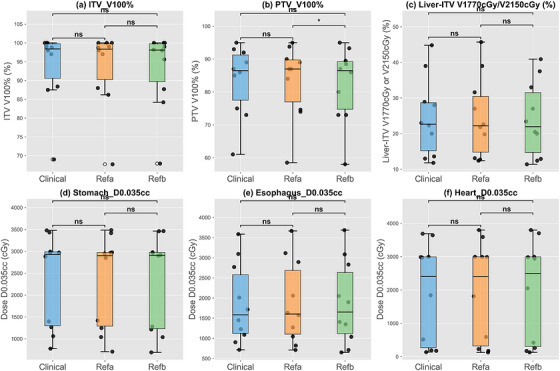
Comparison of key plan quality metrics for Clinical and experimental (Refa and Refb) plans in liver cases. Metrics are presented as (a) ITV V100%, (b) PTV V100%, (c) liver—ITV V21.5 Gy for five‐fraction plans and V17.7 Gy for 3‐fraction plans, and (d‐f) D0.035 cc to the stomach, esophagus, and heart. The connecting lines between the boxplots indicates pairwise *t*‐tests, with “NS” denotes no statistically significant difference (*p* > 0.05).

Figures 6 and 7 show the comparison of various key plan quality metrics for Clinical and experimental (Refa and Refb) plans in prostate and liver cases. The connecting lines between the boxplots indicates pairwise *t*‐tests, with “NS” denotes no statistically significant difference (*p* > 0.05). For prostate cases, target coverage was comparable across Clinical, Refa, and Refb plans. Maximum point 0.035cc doses were similar for urethra, rectal wall and bladder wall, with volumetric constraints V24 Gy < 50% for rectum and V18.3 Gy < 18% for bladder also comparable. All differences were not statistically significant (*p* > 0.05). Similar trend was observed for Liver cases. ITV V100% and PTV coverage were comparable. Liver‐minus‐ITV volumetric constraints (V1770cGy/V2150 < 67%) and maximum point 0.035cc doses to stomach, esophagus, and heart were also similar across plans. No statistically significant differences were observed for any metric (*p* > 0.05). Overall, Clinical, Refa, and Refb plans provided comparable target coverage and OAR sparing for both prostate and liver cases, confirming equivalent dosimetric quality across planning strategies. Detailed Dosimetric comparison tables were provided in appendix.

**FIGURE 8:**
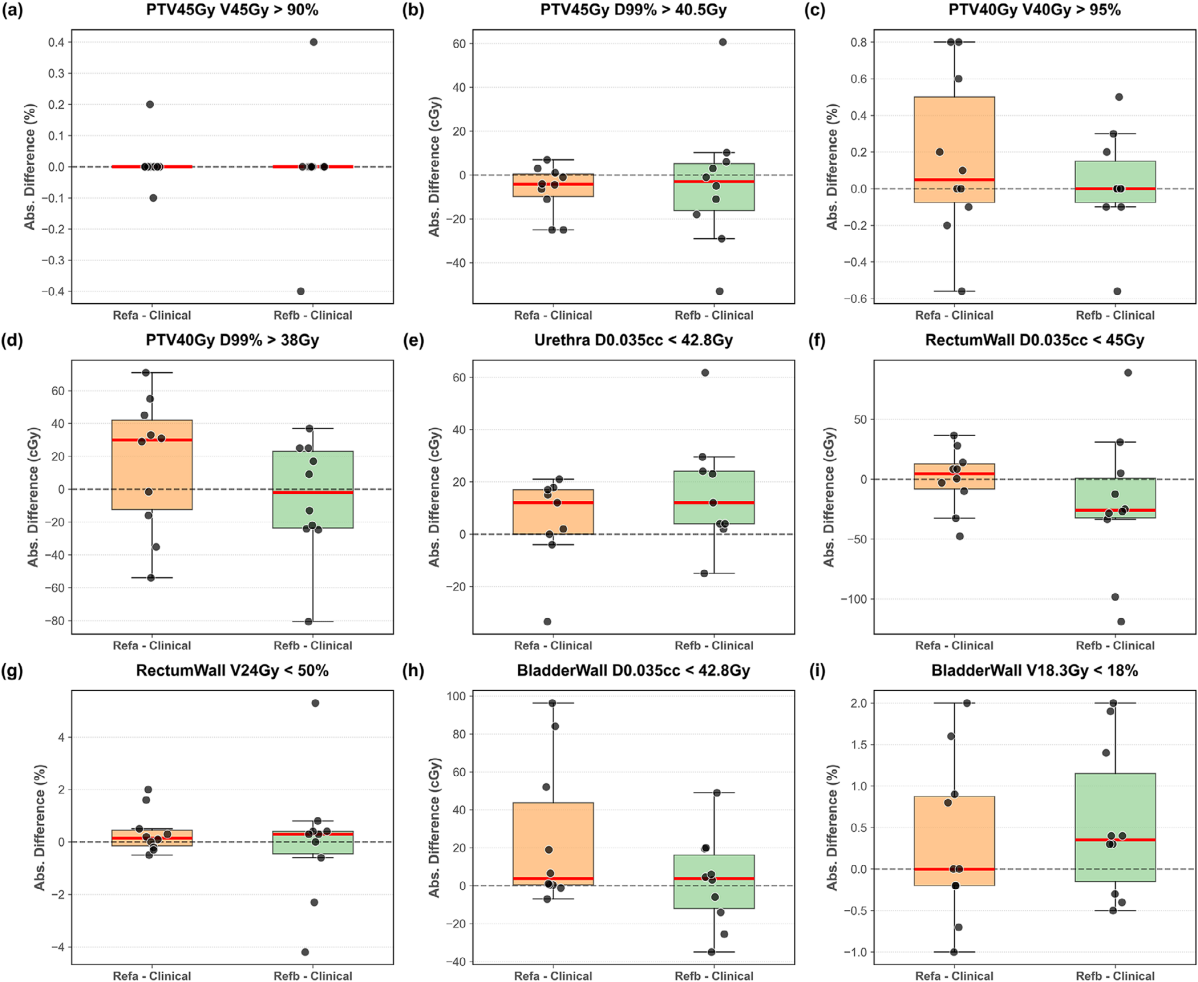
Pair‐wise dosimetric comparison of experimental plans versus clinical plans of prostate cases. Boxplots illustrate the absolute difference in target coverage and OAR sparing for (a–d) PTV parameters and (e–i) critical organ constraints. Orange and green boxes represent the absolute deviation of Refa and Refb from the Clinical plans, respectively. The red line indicates the median difference, individual points represent unique patients.

**FIGURE 9:**
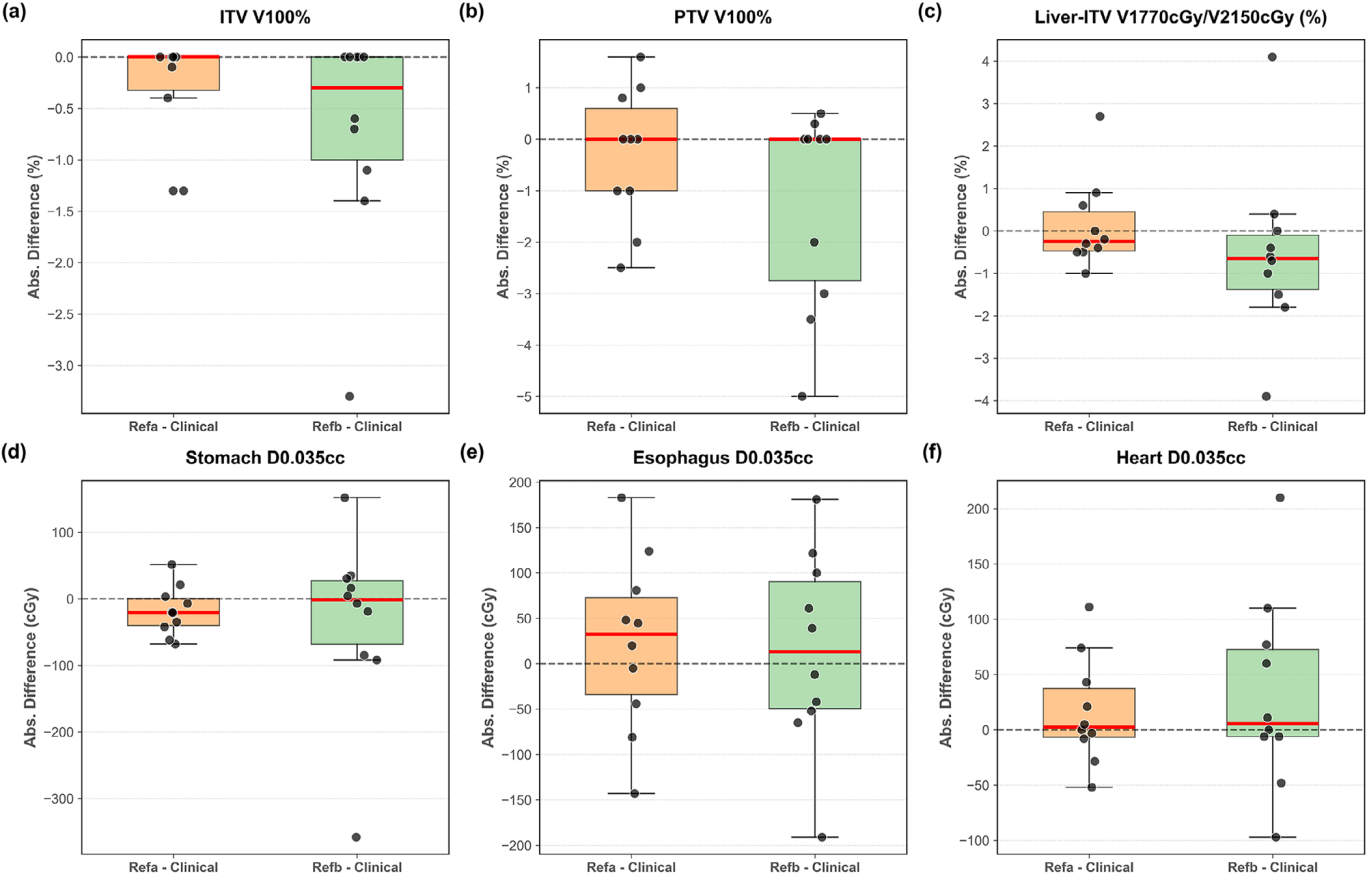
Pair‐wise dosimetric comparison of experimental plans versus clinical plans of Liver cases. Boxplots illustrate the absolute difference in target coverage and OAR sparing for (a) ITV V100%, (b) PTV V100%, (c) liver—ITV V21.5 Gy for 5‐fraction plans and V17.7 Gy for 3‐fraction plans, and (d–f) D0.035 cc to the stomach, esophagus, and heart. Orange and green boxes represent the absolute deviation of Refa and Refb from the Clinical plans, respectively. The red line indicates the median difference; individual points represent unique patients.

Figures 8 and 9 illustrate the pairwise dosimetric differences between experimental (Refa and Refb) and clinical plans. For prostate plans, the absolute maximum dose differences for OARs (urethra, bladder, and rectum) were all below 100 cGy, with the majority under 50 cGy compared to the clinical plans. Deviations were slightly larger for liver cases, likely due to the complexity of managing multiple lesions within the optimizer. While individual cases exhibit larger fluctuations for some dosimetric criteria (e.g., ∼5% in liver sparing) due to the inherent stochastic nature of the optimization process, these variations are not statistically significant across the cohort (*p* > 0.05). Such individual case differences could be further minimized through intensive manual adjustment of IMRT constraints.

### 3.4 Online adaption comparison

**FIGURE 10:**
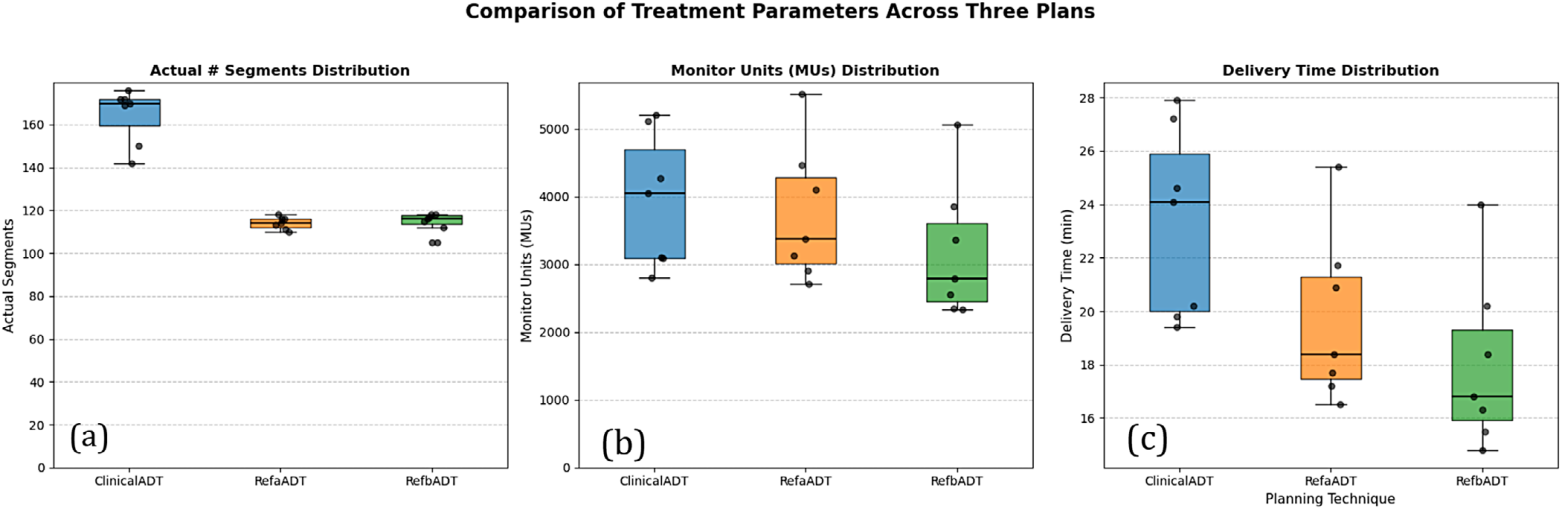
Plan delivery efficiency metrics comparison among all the adaptive plans using the three reference plans. (a) Actual number of segments distribution. (b) Monitor units distribution, and (c) Estimated delivery time (min) distribution.

The online optimization time is dependent on the covering physicist's expertise and confidence in rapidly adjusting IMRT constraints and weights, as well as their judgment on when to terminate the process to achieve both convergence and acceptable plan quality. In our study, all adaptive plans generated from Refa and Refb were consistently completed within 5–13 min, which is in line with our standard clinical optimization timeframe. For consistency, all adaptive plans were normalized to the target coverage of the corresponding clinical adaptive plan. The comparisons of delivery efficiency metrics are shown in Figure 10. All the adaptive plans were able to meet the clinical dosimetric goals. A similar trend was observed for the online adaptive plans as in initial reference plans: RefaADT and RefbATD reduced delivery times by 1.9–6.1 min for prostate cases (including ATP, ATS, micro‐boost, and whole‐prostate boost scenarios) and by 2.5–7.8 min for the liver cases evaluated.

## 4 DISCUSSION

This study demonstrates that adjustment of planning parameters can markedly improve delivery efficiency in MR‐Linac adaptive radiotherapy without sacrificing target coverage or organ‐at‐risk protection. Our results demonstrate practical strategies to balance plan quality and deliverability efficiency—particularly for online adaptive on Unity, where treatment time is a critical bottleneck. These findings provide a framework for parameter optimization that enhances workflow efficiency, maximizes system utilization, and supports the broader clinical adoption of high‐quality adaptive treatments. Based on our single institutional experience, we offer practical recommendations to guide parameter selection and facilitate the effective use of Unity in broader community.

While this study focused on three key planning parameters—maximum number of segments, fluence map smoothing, and minimum segment width—the optimization algorithm includes many additional parameters that can be adjusted on a patient‐specific basis to balance dosimetric quality and delivery efficiency. For example, Minimum Segment Area (MSA) and Minimum MU per Segment also play a role, as increasing these values reduces the number of small, inefficient segments, thereby lowering total MU and delivery time. These parameters were not included in this study, as doing so would significantly expand its scope and complicate reporting. Our goal was to provide practical recommendations: the leaf sequencing parameters used for Refa plans (minimum segment width = 0.5 cm, minimum segment area = 2 cc, minimum MU per segment = 4 MUs, and a maximum of 120 segments) were found to be reasonable, maintaining clinically acceptable plan quality and stability during online adaptation for both prostate and liver cases.

However, increasing the minimum segment width is not universally applicable. This approach is effective primarily for cases with larger targets and simpler geometries. For cases involving small targets, irregular shape, or targets adjacent to (or overlapping with) critical OARs, increasing the segment width can lead to plan quality deterioration, slower optimization convergence, and difficulty achieving adequate target coverage. These complex geometries inherently require finer modulation, necessitating smaller segments to conform the dose while sparing critical structures. Conversely, overly small segments can compromise deliverability and dosimetric accuracy. Segments with very few MUs (< 3 per control point) can challenge MLC motion control and may produce anomalies due to communication lag between the MLC system and the linear accelerator console. In our clinic, the default minimum MU per segment is 4 MUs, though this value is sometimes increased—for example, in large liver cases—to improve delivery efficiency and stability.

Each institution may either reference these parameter values as a starting point—particularly if their planning protocol is similar to ours—to streamline implementation and reduce trial‐and‐error or apply the same optimization strategy described in this work to identify their own key parameters based on local planning practices and clinical priorities.

## 5 CONCLUSION

Systematic adjustment of Unity IMRT planning parameters—specifically limiting total segment number, minimum segment width, and fluence smoothing—can markedly improve delivery efficiency while maintaining clinical acceptable dosimetric quality. These findings provide practical, evidence‐based guidelines for parameter selection in Unity planning, supporting reduced treatment times, improved patient throughput, and broader clinical feasibility of MR‐guided online adaptive radiotherapy.

## AUTHOR CONTRIBUTIONS

**Ying Zhang**: Conceptualization; methodology; data curation; formal analysis; writing—original draft; writing—review & editing. **Shanshan Tang**: Data curation; formal analysis; writing—review & editing. **Christopher Kabat**: Data curation; writing—review & editing. **Justin Visak**: Data curation; writing—review & editing. **Tsuicheng Chiu**: Technical support; writing—review & editing. **Ruiqi Li**: Data curation. **Steve Jiang**: Project administration; writing—review & editing. **Mu‐Han Lin**: Methodology; writing—review & editing.

## CONFLICT OF INTEREST STATEMENT

The authors have nothing to report.

## Supporting information



Suppoting Information
