# Supplementary material for: Strategies for enhancing delivery efficiency on MR‐Linac: A dosimetric study and historical plan review
Source: J Appl Clin Med Phys. 2026 May 27;27(5):e70638. doi: 10.1002/acm2.70638 (PMC13239387; doi:10.1002/acm2.70638)
Supplement: Supplementary file 1 — Suppoting Information [file ACM2-27-e70638-s001.docx]

Supplemental materials

Table S1: Summary of plan characteristics of the dosimetric study

Table S2: Summary of the delivery efficiency metrics of the initial reference plans and the adaptive plans.
